# Integrating Polypropylene Fibers and Cement in Clays for Sustainable Clay Bricks

**DOI:** 10.3390/polym17243244

**Published:** 2025-12-05

**Authors:** Muawia Dafalla, Awadh Abden

**Affiliations:** Department of Civil Engineering, College of Engineering, King Saud University, Riyadh 11421, Saudi Arabia; 438106612@student.ksu.edu.sa

**Keywords:** clay bricks, polypropylene, cement, fibers, plastic clay, strength

## Abstract

This study investigates how adding polypropylene fibers and cement affects the strength of highly plastic clay used in clay bricks. The research looked at various curing times to improve the strength of clay bricks for effective use in the construction industry. A fiber content of 0.2% was added to the clay and compared to untreated control samples improved with varied amounts of cement (2%, 4%, and 6%). The influence of curing on strength increase was explored, as well as the profile of the stress–strain relationship. The compressive strength increased by 53% to 140% after 7 days of curing, which is almost a quarter of the strength attained after 28 days. The results showed a considerable increase in strength, illustrating the cumulative benefits of longer curing times and the suggested additions. Fiber addition was shown to be associated with a significant increase in compressive strength. This advantage is due to the particle connection established by incorporating the fibers and cement into the mixture. Improvement in tensile and shear strength was investigated. It was also found that fibers made the material more ductile. It was noted that using cement alone can increase the compressive strength but cracking and shrinkage control may not be achieved. When compared to the untreated sample, mixtures containing 0.2% fibers and treated with 2%, 4%, and 6% cement increased compressive strength by 225%, 390%, and 630%, respectively. This improvement is comparable to a 2-, 4-, or 6-fold improvement. This increase will enhance the supporting capacity of the non-load-bearing clay bricks.

## 1. Introduction

Polypropylene fiber (PP) is synthesized by polymerizing propylene molecules, resulting in a material with low density and high tensile strength. Polypropylene fibers have evolved as a useful thermoplastic polymer in recent decades. Advancements in the manufacture and application of PP have led to its widespread use across various industries. This group includes, but is not limited to, the concrete and geotextile industries. This study considers adding polypropylene fibers to enhance the strength and durability of air-dried clay bricks.

The use of air-dried clay bricks in construction is a traditional practice of homes with efficient thermal properties. Clayey soils, particularly those rich in highly plastic clay, are prevalent in arid and semi-arid regions. These soils are notorious for their significant volume changes with variations in moisture content, which pose substantial challenges in construction activities, particularly for structures that depend on stable foundations. The shrink-swell behavior of highly plastic soils can lead to cracking, structural damage, and increased maintenance expenses, necessitating effective stabilization methods to enhance their engineering properties and ensure the durability and safety of constructions.

One innovative approach to utilizing clayey soils in construction is the production of building bricks formed from them with enhanced properties through the addition of cement and fibers. Historically, clay brick masonry has been one of the most enduring construction techniques, with evidence of its use dating back to ancient civilizations like Mesopotamia and Egypt [1]. We must increase the compressive strength of clay bricks to improve their structural function and extend their lifespan in buildings. To overcome drawbacks including low strength and significant water absorption, recent studies have concentrated on adding cement and polypropylene (PP) fibers as additions to conventional clay bricks.

The ability of clay bricks to withstand various environmental challenges, coupled with their availability, makes them an attractive option. However, traditional clay bricks often lack sufficient compressive strength and durability when subjected to the unique conditions presented by highly plastic soils.

Cement stabilization has emerged as a popular method to improve the mechanical characteristics of soft and expansive clays. This technique offers rapid formation and adequate plasticity, significantly enhancing compressive strength [2]. However, cement-stabilized soils typically exhibit low tensile and flexural strengths, which can result in cracking and structural failure under load [3]. To address these limitations, recent research has focused on the incorporation of polypropylene fibers into cement-stabilized soils. Polypropylene fibers are lightweight, have high tensile strength, and require low energy for production, making them an effective additive for improving the performance of cement-stabilized soils [4].

According to studies, adding cement to clay bricks at different amounts, usually between 4% and 14%, can increase their compressive strength by up to 65% when compared to non-stabilized bricks. The longer the curing time, the stronger the material gets. After 28 days, it is 52% stronger than after 14 days [5,6].

Numerous investigations have validated the benefits of polypropylene fiber reinforcement in cement-stabilized soils. For instance, Kaniraj and Havanagi (2001) and Tang et al. (2007) [7,8] highlighted significant improvements of Portuguese soft soils that were solidified with polypropylene fiber-reinforced blast furnace slag. Similarly, Xiao et al. (2017) [9] established a predictive model for tensile strength in polypropylene and polyvinyl alcohol fiber-modified cement-stabilized clay, revealing the influence of fiber length and content on performance.

In consideration of international standards, the compressive strength of masonry units is a critical parameter. According to ACI [10], the minimum compressive strength for masonry units typically stands at 1500 psi (10.3 MPa), while the British Standard [11,12] classifies bricks with minimum strengths ranging from 35 N/mm^2^ (5000 psi) for Class C to 75 N/mm^2^ (10,000 psi) for Class A (British, 1985; ACI, 2005) [10,12]. The level of strength specified by the standard is related to modern frame structures and concrete buildings. However, well-engineered clay bricks that have been systematically reinforced with cement and fibers could make construction in areas with a lot of highly plastic clayey soils more durable, stronger, and better overall.

Research has shown that the compressive strength of clay bricks is closely related to their physical and chemical properties, such as porosity and level of exposure to temperature, which are critical for determining their durability [2,13]. Despite the importance of mechanical properties, few studies have focused on these aspects, particularly regarding the compressive strength of clay bricks, which varies widely [3,14].

This paper aims to investigate the feasibility of using highly plastic clay soil to create high-performance parasitic bricks through the addition of cement and polypropylene fibers. By analyzing the mechanical properties and durability of these bricks, this study contributes to the discourse on sustainable building materials, emphasizing the potential of local resources to improve construction practices in regions where highly plastic clay is abundant. Through this research, we seek to establish a viable alternative to conventional clay bricks, ultimately advancing the field of sustainable construction in challenging environments.

## 2. Materials and Methods

### 2.1. Materials

The clay material from Al-Qatif town, located in the Eastern province of Saudi Arabia, was used in this study. The clay material was placed in an oven at 105 degrees Celsius for 24 h. Al-Qatif clay is classified as highly plastic clay (CH) in accordance with ASTM D2487 [15]. This clay is well known for its aggressive swell and shrinkage properties [16,17]. Several research studies were performed to characterize and evaluate this type of clay [18,19,20]. The standard Proctor maximum dry density is measured at 11.5 to 12 kN/m^3^ [21]. The optimum moisture content is reported as 30 to 32%. Al-Qatif clay has a liquid limit ranging from 130 to 150 and a plasticity index between 60 and 70, exhibiting relatively higher swell phenomena and sensitivity to the direction of sampling.

In this research, we utilized Portland cement Type I from Al Yamama Company (Dammam, Saudi Arabia), which adheres to the rigorous standards set by ASTM C150 [22] as well as the regulations established by the Saudi Arabian Standards Organization (SASO). This cement type is widely recognized for its consistent quality and suitability for various construction applications, making it an ideal choice for our study.

For the fiber reinforcement, we incorporated polypropylene fibers measuring 12 mm in length, sourced from Propex Operating Company, LLC, based in Ringwood in the UK. These polypropylene fibers are characterized as fine monofilaments, designed to enhance the mechanical properties of cement-stabilized composites.

**Figure 1:**
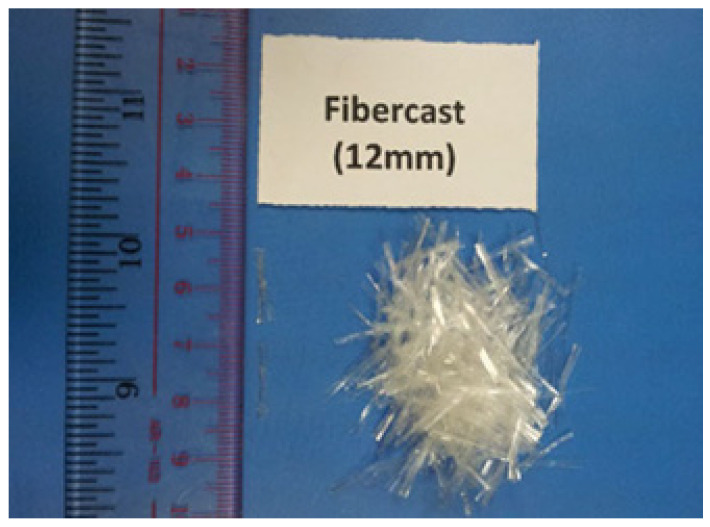
Fibercast 12 mm used in this study (Dafalla and Moghal, 2016) [23].

The polypropylene material exhibits remarkable thermal stability, with a melting point of 162 °C (324 °F) and an ignition point of 593 °C (1100 °F), which underscores its suitability for construction applications where temperature fluctuations may occur. The fibers also have low electrical and thermal conductivity, which can help the final composite product insulate better (Figure 1, Table 1).

Dafalla and Moghal [23] stated that the linear shrinkage tested for two clayey soils reinforced with 2% polypropylene 12 mm fibers was reduced from 12.63 and 13.76 to 11.47 and 12.42, respectively.

These fibers are light but strong enough to strengthen cement matrices. They have a specific gravity of about 0.91. The guidelines set forth in ASTM D 2166 [24] were meticulously followed throughout the experimental process, ensuring that our methodology aligns with established standards for testing the physical and mechanical properties of the materials used. This high-humidity condition was necessary for the cement to hydrate, which helped the soil–cement matrix bond and get stronger, promoting their potential use in various construction applications.

The clay sample is split into three portions, the fibers are subdivided into three doses, and each portion is hand mixed until uniform distribution is visually attained. Then the three portions are mixed together. The number of portions is not necessarily three and using more will provide better uniformity.

Each sample was meticulously prepared using a mold with inner dimensions of 35 mm in diameter and 70 mm in height. To ensure uniform density throughout the samples, a static compaction method was employed, utilizing two piston rods—one positioned at the top and the other at the bottom of the mold. This approach facilitated even distribution of pressure during compaction, which is critical for achieving consistent mechanical properties in the final product. The compaction rod weighs 0.5 kg and falls over a 100 mm distance. The required compaction is achieved when the computed soil weight is confined within the specified volume.

The samples were designed to achieve a target dry density of 12 kN/m^3^. This specification reflects our objective of optimizing the material’s performance characteristics. Following the preparation, the samples were subjected to a controlled curing process. They were kept in a humidity-controlled environment to maintain a relative humidity of 95%. This high-humidity condition was essential for the hydration process of the cement, promoting effective bonding and strength development within the soil–cement matrix. The tests were conducted at a room temperature of 22 degrees Celsius.

**Figure 2:**
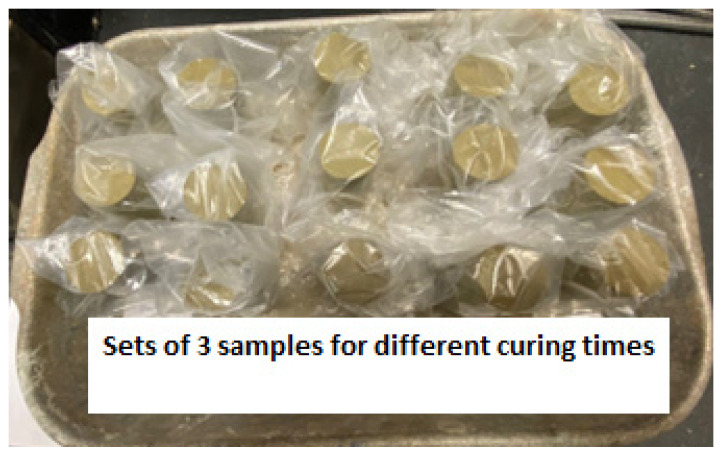
Prepared core samples covered by polythene wraps during curing time.

Curing durations of 7, 14, and 28 days were systematically implemented to assess the evolution of the material’s strength and other properties over time (Figure 2). This extended curing period allows for a comprehensive evaluation of the effects of time on the mechanical performance of the samples, providing valuable insights into the durability and applicability of the soil–cement–fiber composites in construction. By carefully controlling both the density and curing conditions, this study aims to establish a robust foundation for understanding the behavior of these innovative building materials.

### 2.2. Testing Procedure

The investigation program was designed to evaluate the effects of cement and fiber reinforcement on the properties of Al-Qatif clay. To achieve this, three distinct sets of samples were prepared in cylindrical molds, each aimed at exploring different reinforcement strategies and curing durations.

The first set of samples consisted of Al-Qatif clay reinforced with polypropylene fibers. A constant fiber dosage of 0.2% was applied based on the dry weight of the clay. This specific dosage was selected based on previous findings indicating optimum improvements in cohesion at this dose. Dafalla and Moghal, 2016 [23] have shown that while the addition of fibers generally enhances cohesion, increasing the fiber dosage beyond 0.2% can lead to diminishing returns. Specifically, higher dosages (0.4% and above) tend to create discontinuities within the material, particularly at the planes of fiber distribution, which can negatively impact cohesion. Therefore, the 0.2% dosage was deemed optimal for maximizing the reinforcing effects of the fibers without introducing potential weaknesses.

The cement was added in three different dosages: 2%, 4%, and 6%, also based on the dry weight of the clay. To see how different curing times affect the strength and durability of the fiber-reinforced clay, each sample was cured for 7, 14, or 28 days. This systematic approach allows us to investigate the synergistic effects of fibers and cement over time, revealing the optimal conditions for enhancing the performance of the clay–fiber composite.

The second set of samples focused solely on the effects of cement stabilization without the addition of fibers. For this set, the same cement dosages of 2%, 4%, and 6% were utilized, applied to the dry weight of Al-Qatif clay. All of the samples in this set were cured for the same amount of time, which was 28 days. This comparison is essential for understanding the performance of cement-stabilized clay independently of fiber reinforcement, thus providing a clear baseline for evaluating the effectiveness of fiber additions in the first set.

The third set included pure Al-Qatif clay samples, which served as a control group. These samples were prepared without any cement or fiber additions and were cured for 0 days. This baseline data is crucial for understanding the inherent properties of the clay and serves as a reference point against which the performance of the reinforced samples can be measured.

### 2.3. The Tensile Strength of Fiber-Reinforced Clay

Testing tensile strength is generally conducted using the Brazilian test method (ASTM D3967). Each sample was carefully assembled in a mold with inside dimensions of 50 mm and a length of 100 mm. To achieve a homogeneous density, static compaction was used using two piston rods, one at the top and the other at the bottom. The samples were prepared at a dry density of 12 kN/m^3^ and cured for 7 days under constant humidity conditions. Samples were loaded via a spacer onto a flat surface. The machine is outfitted with a calibrated proving ring and displacement dial gauge. The failure tensile strength T is computed using the following formula:

T = 2P/π DL(1)

where P = applied load. D is the specimen’s diameter; L is the length.

It needs to be noted that L/D ratio equal 2 may have an effect on the test results. If correction is necessary, applicable codes need to be considered.

Four samples from Al-Qatif clay, reinforced with different doses of fibers (0, 600, 900, and 1200 gm) by dry weight, were prepared in cylindrical molds as described above to assess the tensile capacity improvement provided due to fiber reinforcement.

Clay has extremely low tensile strength, which is rarely considered in practice. It is highly changeable and depends on the structure, packing condition, and soil suction.

Each mix was prepared in two samples, and the results were averaged. To see the profile of the stress–strain relationship of fiber-reinforced clay, the tensile strength was measured at fixed intervals while the strain rate was kept at 0.3 mm/min.

### 2.4. Shear Tests

The direct shear tests were conducted in accordance with ASTM D3080M-11 [25]. Normal stress levels applied to each sample in a direct shear test for clays enhanced with fibers were 50, 100, and 150 kPa. For this study 0.2% fibers were considered for investigating the effect of fibers on the cohesion and the angle of internal friction. The shear strength of cement–fiber-enhanced clay was taken as half the compressive strength.

### 2.5. Shrinkage Tests of Polypropylene Clay–Cement Mixtures

The shrinkage test method followed was based on the linear shrinkage, but the mold selected was taken as circular (Tex-107-E, 1999) [26]. To assess the shrinkage, clay–cement mixtures were prepared in rings of 70 mm diameter and 20 mm in height with different clay–cement proportions and polypropylene fiber doses; 0, 600, 900 and 1200 gm/m^3^. The rings were placed in an oven of 50 °C for 7 days or until shrinkage stopped. Soil paste is prepared at the liquid limit moisture content; it is then left to dry out in an oven. The wet sample’s initial diameter (D_0_) was measured and compared to the reduced final sample diameter (D_1_) using the following equation:

Linear Shrinkage = (D_0_ − D_1_) × 100/D_0_ (2)

For circular samples, this provides the diameter reduction as a percentage, which is a reliable indicator of linear shrinkage.

### 2.6. XRD and SEM Characterization

**Figure 3:**
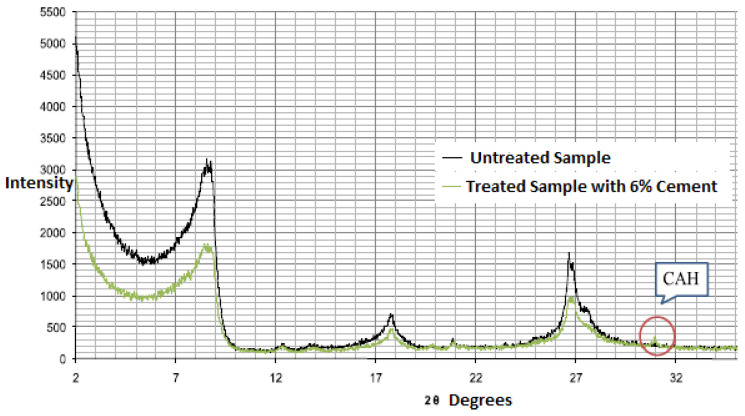
The X-Ray diffraction of Al-Qatif clay, treated and untreated with cement material.

The first author conducted XRD testing for the same material treated with 6% cement added (Mutaz and Dafalla) [27], where peaks of clay minerals can be detected. Calcium aluminate hydrate (CAH—3CaO·Al_2_O_3_·Ca(OH)_2_·12H_2_O) normally forms when adding cement. Figure 3 presents the intensity profile against 2θ.

**Figure 4:**
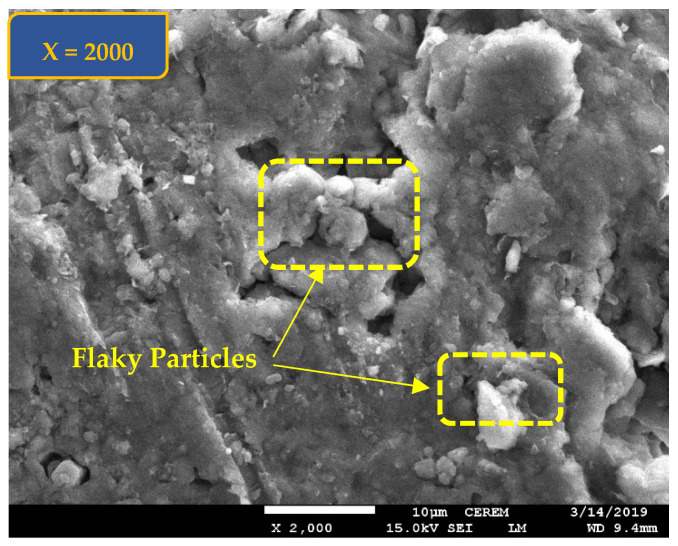
SEM at a magnification of ×2000 for Al-Qatif untreated clay.

**Figure 5:**
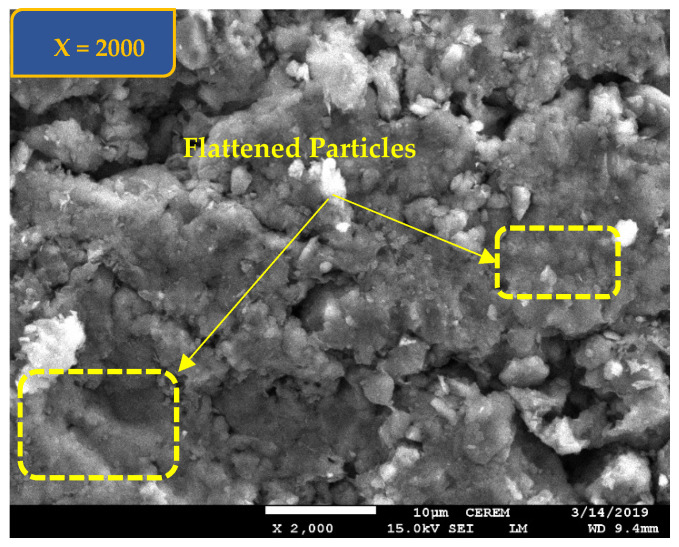
SEM at a magnification of ×2000 for Al-Qatif clay treated with 2% cement.

Scanning electron microscope, SEM, views (Figure 4 and Figure 5) at magnification of ×2000 is also presented for Al-Qatif clay treated by 2% cement (Shaker and Dafalla [28]).

## 3. Results and Discussion

### 3.1. The Compressive Strength

The polypropylene fiber and clay matrix bonding is poor. Bonding using a cementing agent is required to create a satisfactory interlock between particles in clay brick mortar. The introduction of cement was found to be a better solution to bonding and increasing the compressive strength.

**Figure 6:**
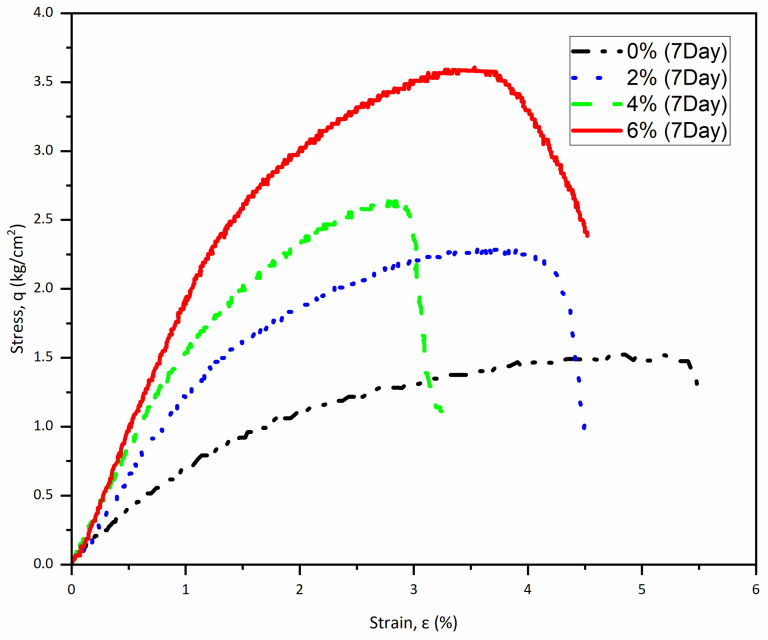
Stress–strain curve of specimens (0.2% Fiber) at 7 days curing.

The results obtained from this study reveal a significant improvement in the compressive strength of samples treated with cement and fibers, either in combination or solely with cement, over time. After a curing period of seven days, the compressive strength of the samples treated with cement that contains 0.2% fibers and 2% cement was measured as 2.3 kg/cm^2^. For 4% and 6% cement, the compressive strength increased to 2.6 and 3.6 kg/cm^2^, respectively. In contrast, the untreated sample, which contained no additives, exhibited a compressive strength of only 1.5 kg/cm^2^. Table 2 summarizes the compressive strengths and the average modulus of elasticity for the tested samples after seven days of curing with a polypropylene fiber content of 0.2%. Figure 6 illustrates the stress–strain curve for these samples.

**Figure 7:**
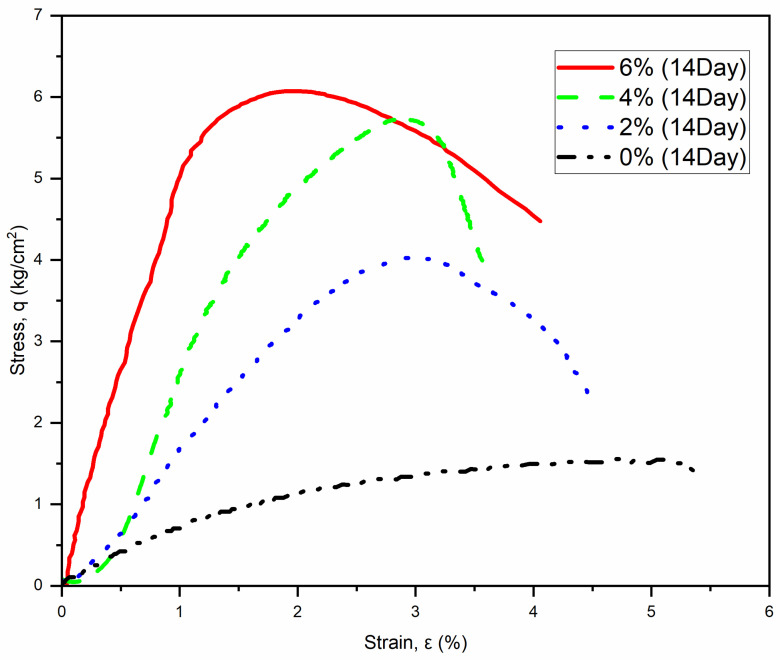
Stress–strain curve of specimens (0.2% Fiber) at 14 days curing.

The improvement in strength became even more pronounced after 14 days of curing. The compressive strength values increased to 4.07 kg/cm^2^ at 2% cement, 5.74 kg/cm^2^ at 4% cement, and 6.11 kg/cm^2^ at 6% cement. Table 3 provides a summary of the compressive strengths and the average modulus of elasticity for the samples tested after 14 days of curing at the same fiber content of 0.2%. Figure 7 presents the stress–strain curve for these samples.

**Figure 8:**
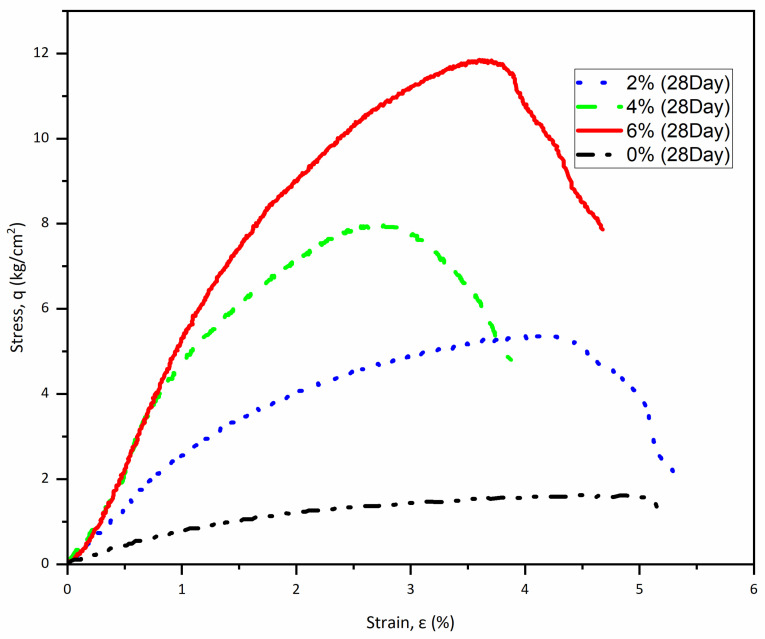
Stress–strain curve of specimens (0.2% Fiber mesh) at 28 days of curing.

By the end of the 28-day curing period, the enhancements in compressive strength were remarkable. The compressive strength values rose to 5.3 kg/cm^2^ at 2% cement, 7.97 kg/cm^2^ at 4% cement, and 11.84 kg/cm^2^ at 6% cement. Table 4 summarizes the compressive strengths and the average modulus of elasticity for the samples tested after 28 days of curing, at a polypropylene fiber content of 0.2%. Figure 8 illustrates the stress–strain curve for these samples.

**Figure 9:**
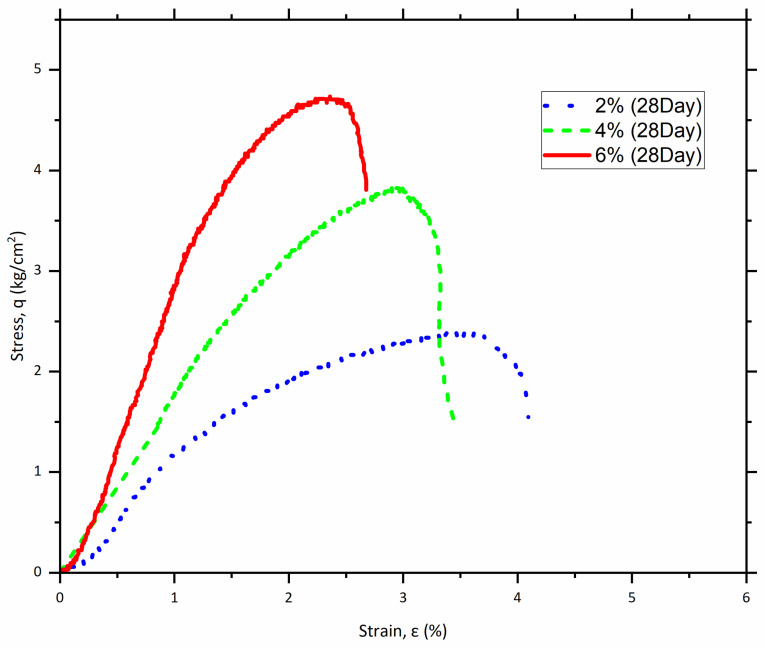
Stress–strain curve of specimens (0% Fiber mesh) at 28 days curing.

Additionally, for the samples with cement and without fibers reported compressive strength values of 2.4 kg/cm^2^ at 2% cement, 3.83 kg/cm^2^ at 4%, and 4.74 kg/cm^2^ at 6% after 28 days curing. Table 5 presents a summary of the compressive strengths and the average modulus of elasticity for these samples, while Figure 9 depicts the corresponding stress–strain curves.

The elastic modulus computed at different curing conditions may not be proportional to the curing time due to the rate of hydration and the clay–water interactions.

In comparing the unconfined compressive strength results across different curing periods and treatment methods, a clear trend emerges. The initial 7-day strength values indicate early gains, but the substantial increases observed at the 14-day and 28-day marks point to extended curing times and the effectiveness of both cement and fiber treatments. For instance, the 2% cement sample increased from 2.3 kg/cm^2^ at 7 days to 5.36 kg/cm^2^ at 28 days; these results represent a remarkable increase of approximately 43%.

Similarly, the 4% and 6% cement samples exhibited significant enhancements, demonstrating increases of 33% and 31%, respectively, when comparing their 7-day and 28-day strengths. In contrast, the untreated control sample consistently showed a low compressive strength of 1.5 kg/cm^2^ throughout the testing period, which starkly contrasts with the treated samples. This study highlights the effectiveness of incorporating cement and fibers in improving material properties. It is important to note that the early development of strength is greater with low cement content. This feature is attributed to the hydration and the area of exposure of cement to water. Thick lumps of cement require more time to reach full hydration.

The analysis also reveals that even when cement was added without fibers, the compressive strength increased significantly. The 2% cement sample reached 2.4 kg/cm^2^, reflecting a −63% increase from the untreated sample. At 4% and 6% cement, the increases were 39% and 32%, respectively. These changes further emphasize the role of cement in enhancing soil properties, regardless of the fiber addition.

Mixtures including 0.2% fibers and treated with 2%, 4%, and 6% cement achieved improvements of 225%, 390%, and 630% increases in compressive strength, respectively, when compared to the untreated sample. This is comparable to 2-, 4-, and 6-fold improvements.

Dafalla and Moghal [23] demonstrated an increase in shear strength in fiber-reinforced clay for different types of polypropylene fibers. Dafalla et al., 2017 [29] also highlighted the enhancement to the tensile strength of plastic clay reinforced by polypropylene fibers. In the presence of a cementing agent, a 100% increase in the tensile strength can be obtained.

**Figure 10:**
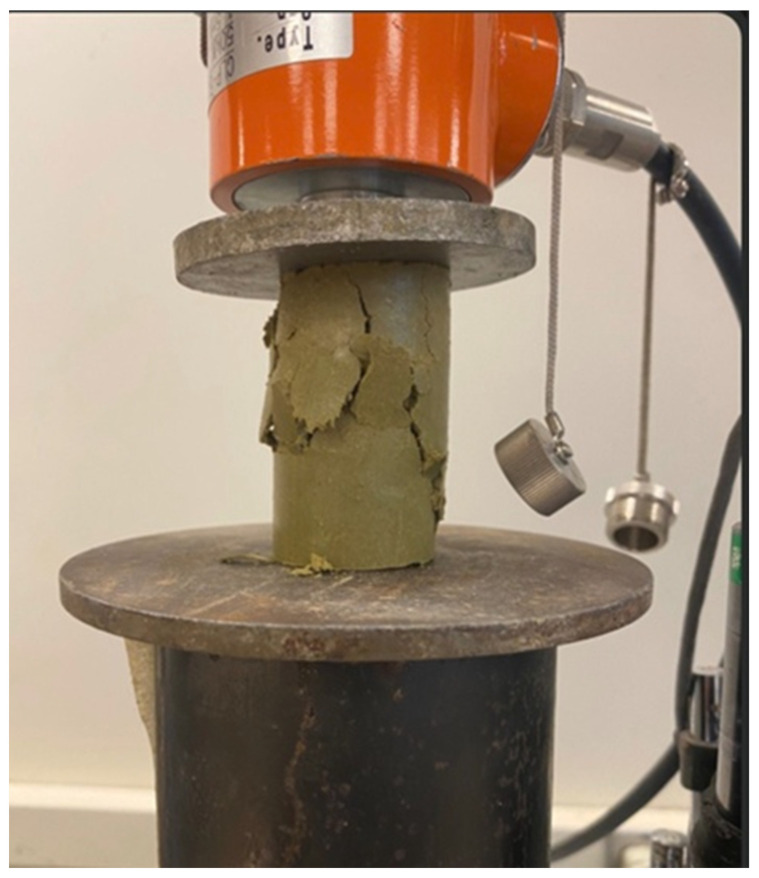
Close-up view of a typical failure under compression.

**Figure 11:**
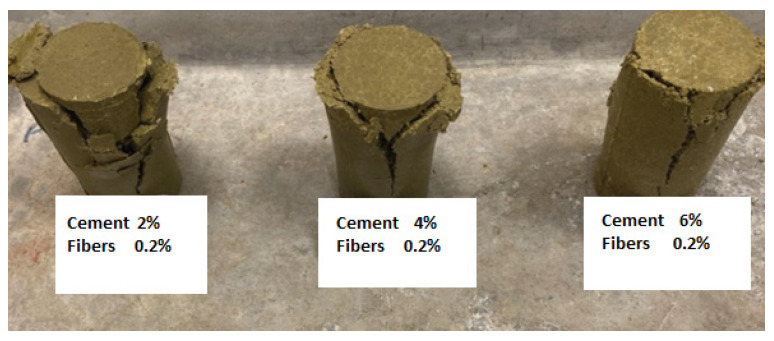
Vertical shear failure planes for 2%, 4%, and 6% cement content with 0.2% fiber-reinforced clay samples.

The mode of failure indicated multiple vertical shear planes (Figure 10 and Figure 11) for all samples tested at 2%, 4%, and 6% cement content.

The addition of fibers increases the structural toughness [30] and the durability in general. When added to the clay bricks, they become less likely to crack and break under force. Moreover, the fibers can enhance control of cracks. Propagation of cracks could compromise durability.

Polypropylene fibers function as micro-reinforcements when added to brick matrices. They increase compressive and flexural strengths by bridging microcracks and more uniformly distributing forces. According to experimental findings, the greatest increase in compressive strength occurs when PP fibers are added at an optimal level that prevents excessive amounts, which could lead to fiber aggregation and weakening of the material.

Fiber-reinforced clay bricks are a viable construction choice due to their increased durability, which provides greater resilience to various environmental and mechanical loads as compared to ordinary clay bricks.

### 3.2. The Results of Tensile Tests

The approach used followed the guidelines outlined in ASTM D3967 [31]. Fiber-reinforced clay bricks are a viable construction choice due to their increased durability, which provides greater resilience to various environmental and mechanical loads as compared to ordinary clay bricks.

**Figure 12:**
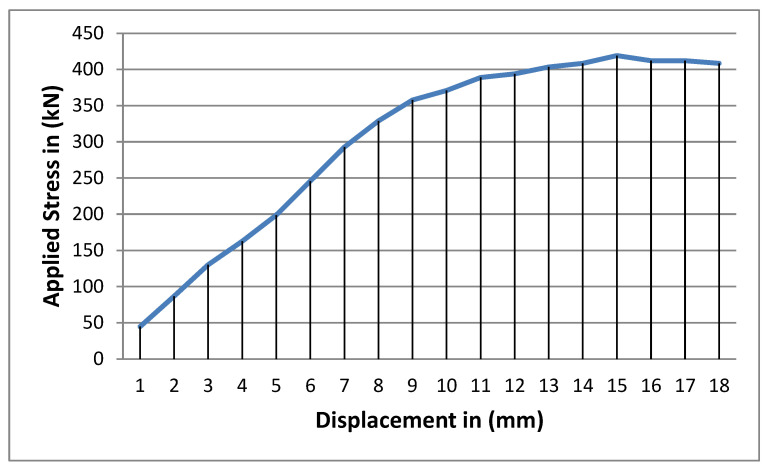
The splitting tensile strength of fiber-reinforced clay with 900 gm/m^3^.

**Figure 13:**
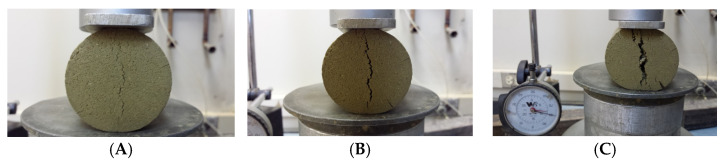
Failure progress in a Brazilian tensile test for clay reinforced by 900 gm/m^3^. (**A**) Central splitting area started; (**B**) splitting progress retarded by fibers; (**C**) failure state reached.

The test results indicated that adding fibers without a cementing agent is unlikely to add significant tensile strength to the clay material for small dosages (Table 6, and Figure 12). The tensile strength reported for clays without reinforcement with fibers was 54.4 kPa. The tensile strength did not increase with the addition of a 600 gm/m^3^ fiber dose and actually decreased with a 900 gm/m^3^. A slight increase is shown with the dose of 1200 gm/m^3^. We can understand the decrease by considering that the fibers may not stretch in a straight line and may not adhere to the clay particle. When adding a cementing agent like cement or lime, a bond can be established. This study suggests that the use of fibers without a cementing agent is of little or no benefit to highly plastic clay and clays in general. Observations on modes of failure indicate a ductile nature, which is facilitated by the fibers. Failure planes started close to the edges before the major splitting failure plane was developed at the central zone of the sample (Figure 13). Testing the tensile strength for the material with cement will only be attributed to the cementing agent. Fibers can act as a crack control, preventing shrinkage and thermal effects.

### 3.3. Effect of Polypropylene Fibers in Shear Strength of Clays

The shear strength of clay reinforced with polypropylene fibers is studied by Dafalla and Moghal [23]. The cohesion was found to increase from 0.8 kg/cm^2^ for non-reinforced clay to 1.1 kg/cm^2^ for clay reinforced with 0.6% polypropylene fiber. The same improvement is reflected in the angle of internal friction, which jumped from 13.7 degrees for non-reinforced clay to 13.7 degrees for clay with 0.6% polypropylene fiber. Direct shear tests were conducted with normal stresses of 0.5, 1, and 2 kg/cm^2^. The major improvement in shear strength comes from the cementing agent rather than the fibers. The results of shear strength for the fiber–cement-reinforced clays are given in Table 7. The data shown indicates that shear strength can increase by 4-fold when 6% cement is considered.

### 3.4. Effect of Polypropylene Fibers and Cements in Shrinkage of Clays

**Figure 14:**
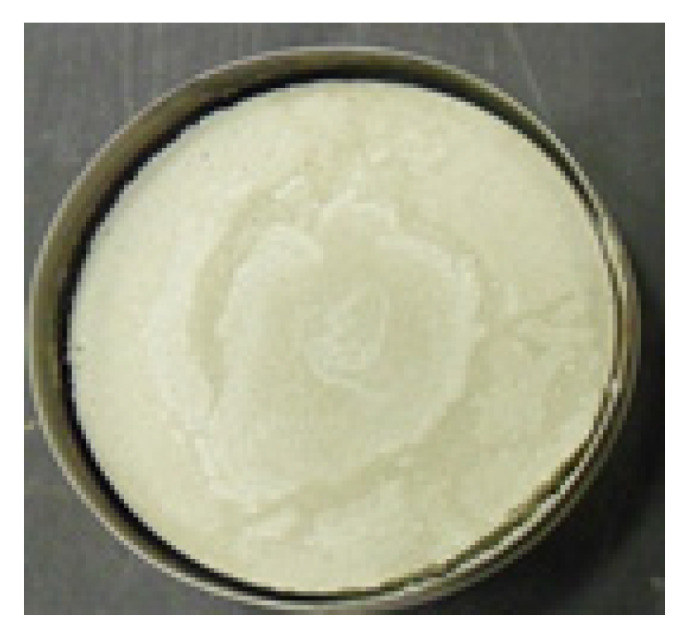
Untreated clay–cement mold.

**Figure 15:**
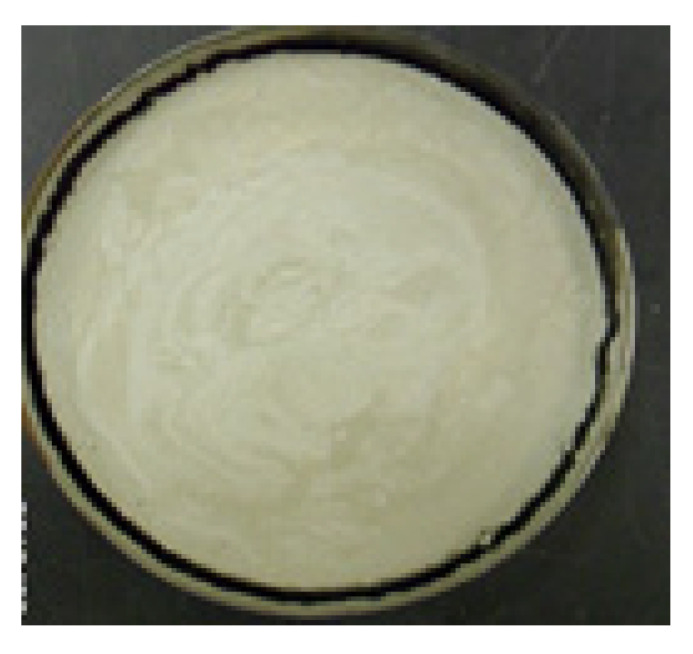
Polypropylene treated clay–cement mold.

The shrinkage and expansion of clay are strongly related to the mixture content. The test results indicated that clay–cement polymer mixtures prepared at different clay–cement contents behave differently with regard to shrinkage. It is reported that the more the clay, the higher the change in mold diameter (Figure 14 and Figure 15). The role of polypropylene fiber was dependent on the fiber content. Optimum fiber content is believed to be ideal in reducing shrinkage. Low fiber content, as 600 gm/m^3^, was found to cause shrinkage to increase for nearly all clay–cement mixtures. The same trend is reported for a fiber-rich mix of 1200 gm/m^3^. The 900 gm/m^3^ was found to be the optimum fiber content for reducing shrinkage and indicated reduction in shrinkage for all clay–cement content tested. The tested fiber length used in this study was 12 mm (Table 8). Table 8 shows the reduction in the diameter measured in (mm) for different fiber content. When a soil or material sample dries from a wet state (generally at liquid limit moisture content) to an oven-dry condition, it experiences linear shrinkage, which is the reduction in one dimension (usually length or diameter). The central part normally stands firm, and the reduction in diameter is shown as peeling off from the circular ring.

The XRD examinations indicated peaks of different intensities for Al-Qatif clay treated with 6% cement and untreated Al-Qatif clay. The mineralogy remained unaffected but a CAH (Calcium aluminate hydrate) peak is observed. This peak indicates the presence of pozzolanic compounds.

Hydrated Al-Qatif clay with 2% cement was shown in SEM of a flatter shape compared to the dry untreated clay indicating a flaky particle nature.

## 4. Further Discussion

### 4.1. Long-Term Evaluation of Polymer–Cement-Enhanced Clay Bricks

The clay bricks treated with polypropylene and cement are expected to have better durability than untreated clay bricks. This improvement is due to the pozzolanic compounds formed as a result of adding cement. Although there is not much work examining the durability of such materials, it can be predicted based on the performance of cement-stabilized clayey soils. The untreated clayey bricks are generally brittle and subject to cracking when exposed to drying or high temperature. The cementing agent provided to the clay brick with fibers adds more strength, while the fibers add more ductility. These improvements can be projected to the durability and the lifetime of such a new product.

Phantachang et al. [32] stated that curing time contributed substantially to strength development, with polymer-modified soil–cement mixtures demonstrating superior performance compared to those without polymers.

Miturski et al. (2025) [33] studied the use of adding polypropylene fibers. They reported that adding only 0.25% fibers increased UCS by about 25% at 4% cement. This is comparable to the findings of this study. Their reinforcement index model further showed that 0.5% fibers could reduce cement needs by roughly 25% for equivalent strength.

The type of clay studied in this research poses serious construction challenges, and the regional stabilization data and methods are valuable. Although other smectite minerals are likely to behave in a similar way. The information provided here represents useful data for this regional clay and helps predict its behavior when stabilized with fiber and cement. This improvement is not limited to the compressive strength but can be seen in improving the ductility and controlling the cracks. The bonded fibers resist tensile strength and stop the development of the cracks. Fibers were also found to drastically reduce the swelling when combined with a cement or lime.

Fiber–matrix bonding helps in forming integrated composite materials that move together until slippage occurs. The stronger the binder, the better the resistance to control cracks and halt the swell. The hydration effects are reflected by the different curing times and cement content. The more cement content, the more time required to achieve the full strength.

The polypropylene fiber improvement methods attracted many researchers in recent years [34,35,36,37], and all of them confirmed the increase in clay strength and the better control of swelling and cracking. It can be stated that the combination of cement and polypropylene can increase the longevity and strength parameters and reduce brittleness and crack potential, which will certainly lead to better mechanical performance over time. The proposed material can be affected by the severe environmental conditions; therefore, it is recommended to limit its application to housing, non-load-bearing structures, or arid region construction.

### 4.2. Results Outcome and Leads for Future Works

Overall, these findings collectively underscore the effectiveness of using cement and fibers in enhancing the mechanical properties of treated soil samples. The progressive increase in compressive strength over the curing durations demonstrates the material’s potential for improving using these two additives. The more time allowed for curing, the more strength gain is expected. The bond between hydrated cement and clay particles is significantly increased with extended drying. Furthermore, the results contribute valuable insights for future research and applications in the construction industry, particularly in contexts where improved structural integrity and durability are essential. This study lays the groundwork for further exploration of optimal treatment methods and material combinations to enhance the performance of soil in engineering applications. Cost-performance analysis of materials and performance improvement can also be investigated to assist practice engineers in selecting appropriate mixtures.

## 5. Conclusions

The findings of this study clearly indicate that the incorporation of cement, both with and without fiber additives, substantially improves the compressive strength of treated plastic clay bricks over time. The marked increases in strength at 14 and 28 days highlight the importance of extended curing durations in achieving optimal material properties. Notably, while the 2% cement sample achieved a remarkable increase, the untreated control material consistently exhibited low strength, emphasizing the effectiveness of treatment methods. Mixtures including 0.2% fibers and treated with 2%, 4%, and 6% cement achieved improvements of 225%, 390%, and 630% increases in compressive strength, respectively, when compared to the untreated sample. This is comparable to 2-, 4-, and 6-fold improvements.

This study indicated that adding polypropylene fibers to highly plastic clay will benefit tensile strength at a particular dosage that needs to be determined based on trials and workability. The bond between the fibers and clay cannot be established without considering a cementing agent. The fiber addition is also found to give the clay a ductile state where large deformation can be reported beyond the elastic or semi-elastic zone. Failure was found typical to Brazilian tests of various materials (ASTM D3967). It is noted that cracking starts at the rim of the sample until a major splitting failure plane is developed at the central zone. Shear strength and shrinkage properties were all found to improve when considering the combined effect of the polypropylene and cement.

The proposed material can be affected by severe environmental conditions; therefore, it is recommended to limit its application to housing, non-load-bearing structures, or arid region construction.

## Figures and Tables

**Table 1:** Physicochemical properties of fibers.

	Fibercast 500
Melt point	324 °F (162 °C)
Ignition point	1100 °F (593 °C)
Thermal conductivity	Low
Electrical conductivity	Low
Acid and salt resistance	High
Tensile strength	440 N/mm^2^
Specific gravity	0.91
Water absorption	Nil
Alkali resistance	Alkali proof

**Table 2:** Unconfined compressive strength σ_7_ (kg/cm^2^) of specimens at 7 days of curing.

Sample ID	Cement (%)	Fiber (%)	σ_7_ (kPa/kg/cm^2^)	E (MPa)
Sample 1	0	0	147.1/1.5	3.1
Sample 2	2	0.2	225.5/2.3	6.1
Sample 3	4	0.2	255.0/2.6	11.0
Sample 4	6	0.2	353/0/3.6	10.6

**Table 3:** Unconfined compressive strength σ_14_ (kg/cm^2^) of specimens at 14 days curing.

Sample ID	Cement (%)	Fiber (%)	σ_14_ (kPa/kg/cm^2^)	E (MPa)
Sample 1	0	0.0	153.0/1.56	3.36
Sample 2	2	0.2	399.3/4.07	14.21
Sample 3	4	0.2	562.6/5.74	20.01
Sample 4	6	0.2	599.7/6.11	34.65

**Table 4:** Unconfined compressive strength σ_28_ (kg/cm^2^) of specimens at 28 days curing.

Sample ID	Cement (%)	Fiber (%)	σ_28_ (kPa/kg/cm^2^)	E (MPa)
Sample 1	0	0.0	159.8/1.63	3.66
Sample 2	2	0.2	519.8/5.30	9.83
Sample 3	4	0.2	781.4/7.97	20.15
Sample 4	6	0.2	1161.2/11.84	24.83

**Table 5:** Unconfined compressive strength σ_28_ (kg/cm^2^) of 0% fiber specimens at 28 days curing.

Sample ID	Cement (%)	Fiber (%)	σ_28_ (kPa/kg/cm^2^)	E (MPa)
Sample 1	0	0	159.8/1.63	3.66
Sample 2	2	0	235.4/2.40	5.71
Sample 3	4	0	375.7/3.83	10.90
Sample 4	6	0	464.7/4.74	17.36

**Table 6:** Splitting tensile strength (kPa) for fiber-reinforced clay.

Fiber Amount	Tensile Strength (kPa)
0 g/m^3^	54.4
600 g/m^3^	54.1
900 g/m^3^	53.4
1200 g/m^3^	56.6

**Table 7:** Shear strength for clay enhanced with cement and polypropylene.

Cement (%)	Fiber (%)	Shear Strength (kg/cm^2^)
0	0	0.78
2	0.2	2.04
4	0.2	2.87
6	0.2	3.05

**Table 8:** The reduction in diameter (mm) due to shrinkage of grout for a range of fiber content.

Clay %	0 gm/m^3^ Fiber (mm)	600 gm/m^3^ Fiber (mm)	900 gm/m^3^ Fiber (mm)	1200 gm/m^3^ Fiber (mm)
15%	1.41	1.75	1.20	1.78
30%	2.02	2.71	1.75	2.70
45%	3.09	3.17	2.42	3.48
60%	3.87	4.19	3.27	4.54
75%	4.47	4.2	4.24	4.37
90%	5.5	5.15	5.07	6.05

## Data Availability

The data used to support the findings of this study are included in the tables and figures provided.
